# Effect of Action Observation Training on Spasticity, Gross Motor Function, and Balance in Children with Diplegia Cerebral Palsy

**DOI:** 10.3390/children7060064

**Published:** 2020-06-18

**Authors:** Young-a Jeong, Byoung-Hee Lee

**Affiliations:** 1 Graduate School of Physical Therapy, Sahmyook University, 01795 Seoul, Korea; nay5130@naver.com; 2 Department of Physical Therapy, Sahmyook University, 01795 Seoul, Korea

**Keywords:** cerebral palsy, action observation, spasticity, gross motor function, balance

## Abstract

This study evaluated the effect of action observation training on spasticity, gross motor function, and balance in children with spastic diplegia cerebral palsy. Eighteen children with cerebral palsy participated in this study. The participants were randomized into the action observation training group (*n* = 9) and a control group (*n* = 9). The action observation training group repeatedly practiced the action with their motor skills, while the control group practiced conventional physical therapy. Both groups received 30 min sessions, 3 days a week, for 6 weeks. To confirm the effects of intervention, the spasticity, gross motor function measurement (GMFM), and pediatric reaching test (PRT) were evaluated. The results showed that in the plantar flexor contracture test of both sides, the Modified Tardieu Scale (MTS) of the right side of knee joints, GMFM-B, C, and D were significantly increased between pre- and post-intervention within both groups (*p <* 0.05). PRT was significantly increased between pre- and post-intervention within the both groups (*p <* 0.05), and there was a significant difference between the two groups (*p <* 0.05). These results suggest that action observation training is both feasible and beneficial for improving spasticity, gross motor function, and balance in children with spastic diplegia cerebral palsy.

## 1. Introduction

Cerebral palsy is a non-progressive disorder that affects the development of the brain of fetuses or infants, and presents as limited activity, movement, and postural disorders [1]. Stiff diplegia is a disorder that shows more dysfunction in the lower extremities than the upper extremities [2], while spasticity diplegia is characterized by ununiformed abnormal movements, unstable continuous movements, and large patterns of motion [3]. Cerebral palsy also leads to limits in balance due to muscle weakness in skeletal muscles, excessive reflexes, simultaneous contraction of agonist and antagonist muscles, delayed response of the ankle muscles, and shrink posture [4].

Balance is important for most functional skill movements; this includes the integration of sensory inputs to structure the body’s perception of the center of gravity, and perform appropriate musculoskeletal responses to unexpected movements or to stabilize during moments of instability [4]. However, in cerebral palsy, when balance is affected, it increases compensation usage of the upper extremities, which is followed by restricted movement of the upper limbs. This may cause limitation in the function of the upper extremities [5], performance, and learning activities of daily life, as well as problems in movement and a limitation of social roles and community participation [6].

Spasticity means intermittent or persistently involuntary disordered sensory motor control caused by upper motor neuron lesions [7]. Prolonged spasticity causes abnormal posture, limitation of movement, and limitation and construction of active or passive joint movement [8]. In order to improve the spasticity and balance in children with cerebral palsy, various treatment intervention methods have been used; these include botulinum toxin injection [9,10], anticipatory postural adjustments [11], dynamic ankle-foot orthosis [12], whole-body vibration [13], and extracorporeal shock wave therapy [14]. Recently, a new method of treating upper limb motor deficits using action observation training has been proposed for patients with stroke [15,16] and cerebral palsy [17,18,19]. Action observation training is a cognitive intervention technique that is used to improve and learn exercise skills in sports athletes, the general public, and patients with motor impairments. This training involves using the activity of mirror neurons with excitement characteristics when actually exercising or watching others perform tasks [16]. Various studies on movement observation training have been proposed, but most relate to restoring the upper limb function of stroke and cerebral palsy patients, and studies on the spasticity, gross motor function, and balance in cerebral palsy for movement observation training are insufficient. Therefore, this study aims to contribute to the improvement of rehabilitation in children with spastic diplegia cerebral palsy by verifying the effect of action observation training on the treatment of spasticity, gross motor function, and balance.

## 2. Materials and Methods

The participants of this study were selected from 30 children who were diagnosed with diplegia cerebral palsy and undergoing physical therapy at K-hospital and E-center in Seoul. The specific selection criteria of the study subjects were children between 5 and 11 years old diagnosed with diplegic cerebral palsy, without visual impairment and visual field defects, able to follow the researcher’s instructions, GMFCS (gross motor function classification system) level I–III, and with ankle dorsal flexors and plantar flexors better than poor + in manual muscle test. The parents of the children consented to their participation in this study after the purpose of the study was explained and they were informed that they could withdraw at any time. The exclusion criteria included children with a modified assessment scale (MAS) of 2 or more, children who have not had a seizure in the last 6 months, or those who received botulinum injections 6 months prior to the study. This study was conducted with the approval of the Research Institutional Review Board of Sahmyook University. The objective and the procedures to be performed in the study were fully understood by the subjects, and all participants’ parents provided informed consent for inclusion in the study. Therefore, this study was based on the ethical principles of the Declaration of Helsinki.

The past history of the 30 children who agreed to the study was examined, and other orthopedic or neurological examinations were performed by the attending doctor before treatment. Of the 30 children at K-hospital, 3 children were under GMFCS level III and 1 child had a seizure within the past 6 months. A total of 22 patients were selected, with the exception of 2 with communication disorders and 2 children who had received botulinum injection 6 months prior. The selected 22 children were divided into either the action observation training group (AOT) at K-hospital or the control group E-center, which is a cerebral palsy treatment center nearby K-hospital for the blind, and each group included 11 participants. All subjects picked a go stone with black or white stone from a box containing 22 pieces of stone. The action observation training program was conducted three times a week for 30 min, for a total of 18 times, and general physical therapy was given 5 times/week for 30 min for a total of 6 weeks. One week before training and 1 week after training proceeded the evaluation. In the AOT, two children who could not participate in the experiment due to personal reasons and seizure dropped out of the control group, and two children who could not participate in the experiment due to personal reasons dropped out. Finally, each group included 9 children, and a total of 18 children were included in the experiment.

### 2.1. Action Observation Training

In this study, action observation training (AOT) focused on spasticity of lower extremities, contracture, gross motor function measurement (GMFM), and balance ability. Children with cerebral palsy watched a video on a 42-inch screen, installed 1 m in front of their chairs, while sitting comfortably with their arms resting, but they were not allowed to physically follow the video or move. The model of the video’s motion observation exercise was performed by a therapist who treated the child, and the training video consists of 4 stages that varied by difficulty, and the video of each step was watched for the entire week. The participants watched a video of a task presented by a therapist, and after completing the assignment, they performed the steps, if a step was too difficult to perform, retraining was conducted. The first stage consisted of movements to improve balance in the sitting position, the second stage consisted of sit-to-stand movements, the third stage consisted of standing movements to improve balance, and the fourth stage consisted of walking sideways (Table 1). The viewing time was 15 min, and 5 min of physical training was conducted with the therapist based on the content of the video, after 5 min of watching. In order to enhance the effectiveness of the action observation training, the participants watched the video at a designated time in a quiet place without noise. The children were instructed to concentrate on the video for 1 min intervals to allow for the attention span of children. Entire experiments were conducted by the same investigator from the beginning to the end of the experiment.

### 2.2. General Physical Therapy

Neurodevelopment treatment is a 1:1 treatment between a patient and therapist. The participants received 6 weeks of general physical therapy, 5 times a week, for 30 min each session, according to the treatment schedule of the hospital. The exercise program included lying to sitting position, moving in the sitting posture, sitting and standing up, posture training for learning a normal gait pattern, weight bearing and weight movement training in the straight posture, walking training on the flat floor, and stair walking.

### 2.3. Outcome Measurements

#### 2.3.1. Spasticity of Ankle Joint

In this study, changes in the spasticity was used; ankle stiffness and Modified Tardieu Scale. An electronic joint goniometer (Gemred, China, 2014) was used to measure the ankle stiffness. In the supine position, the examiner extended the knee joint and examined the ankle stiffness in a relaxed state without muscle contraction. After the subject’s heel fixed and dorsi-flexion with manual force as much as possible, it was maintained for 4 s in the end range. A Modified Tardieu Scale (MTS) was used to measure the muscle spasticity; the reliability for children with cerebral palsy was ICC = 0.54–0.95, which is defined as a high reliability [17]. The Tardieu scale can measure muscle spasticity by testing the response of the muscle to stretch at three types of velocity (i.e., slow as possible, speed of the limb segment when falling, and as fast as possible).

#### 2.3.2. Gross Motor Function

The gross motor function measure-88(GMFM) is a tool for measuring and recording changes in an exercise level over time or as a result of treatment; the scores are ordinal on a 4-point grade scale after observing the movements of children with cerebral palsy. The evaluation items were composed of the following: A scale, lying and rolling position with 17 items; B scale, 20 items in the sitting position; C scale, 14 items in the standing position of the instrument and knees; D scale, 13 items in the standing position; and E scale, 24 items, including walking, running, and jumping activities. In cerebral palsy children, the inter-evaluator reliability was ICC = 0.929 and the test-retest reliability was ICC = 0.92–0.99 [18]. The results were compared using the sitting posture (B scale), device and knee standing posture (C scale), standing posture (D scale), and walking, running, and leap activity (E scale).

#### 2.3.3. Balance Function Measurement

The pediatric arm stretch test is a modified version of the functional reach test (FRT) with forward stretch and side stretch in a sitting position. The test-retest reliability of cerebral palsy children was *r* = 0.54–0.88 and the inter-tester reliability *r* = 0.50–0.93 [19]. The side and forward distances of children from the sitting position were measured before and after intervention.

#### 2.3.4. Data Analyses

SPSS ver. 21.0 was used to calculate the mean and standard deviation. The normality of variables was tested using the Shapiro–Wilks test, and all variables were normally distributed. General characteristics of the subjects were provided as descriptive statistics, and independent t-tests were conducted to identify the differences among the groups. Paired t-tests were conducted before and after the action observation training. Statistical significance was assumed when *p <* 0.05.

## 3. Results

The characteristics of the 18 participants who completed the experiment are shown in Table 2. No differences in gender, age, height, weight, GMFCS level were detected between the two groups at baseline.

### 3.1. Spasticity of Ankle Joint

Changes in the spasticity of the study subjects in the two groups were as follows (Table 3). In the right ankle stiffness test, the AOT increased by 6.58° (*p <* 0.05), from 4.00° before training to 10.58° after training, the control group increased significantly by 4.39° and there was no significant difference between the two groups. In the left ankle joint examination of MTS, the AOT showed a significant increase of 6.10° (*p <* 0.05), and the control group showed a significant increase of 4.38° (*p <* 0.05) and there was no significant difference between the two groups. In the evaluation of spasticity right knee joint of MTS, the AOT showed a significant decrease of 2.91° (*p <* 0.05), and the control group showed a significant decrease of 1.09° (*p <* 0.05) and there was no significant difference between the two groups. In the left knee joint MTS evaluation, the AOT showed a significant decrease of 1.86° (*p <* 0.05), while the control group showed no significant difference and there was significant difference between the two groups (*p <* 0.05).

### 3.2. Gross Motor Function

Table 4 shows the changes in the GMFM before and after the intervention. The mean of the GMFM-B items of AOT showed a significantly increased by 5.12%, from 93.33% before training to 98.45% after training (*p <* 0.05), and the control group was significantly increased by 1.85% (*p <* 0.05), and there was no significant difference between the two groups. The mean of the GMFM-C items significantly increased by 6.56% (*p <* 0.05) in the AOT, and the control group also significantly increased by 2.89% (*p <* 0.05), but there was no significant difference between the two groups. Finally, the mean of the GMFM-E items of the AOT showed a significantly increased by 6.51% (*p <* 0.05), and there was significant difference between the two groups (*p <* 0.05).

### 3.3. Balance Function Measurement

The change in dynamic balance between the two groups is shown in Table 5. The right side stretching average of the children’s arm stretch test of AOT was significantly increased by 3.28 cm (*p <* 0.05), and the control group also significantly increased by 1.40 cm, and there was a significant difference between the two groups (*p <* 0.05). The left side stretching average of the AOT significantly increased by 4.08 cm (*p <* 0.05), and that of the control group significantly increased by 1.42 cm (*p <* 0.05) and there was a significant difference between the two groups (*p <* 0.05). The average of the right forward extension of the pediatric extension test significantly increased by 4.60 cm, and the control group significantly increased by 2.02 cm (*p <* 0.05) and there was a significant difference between the two groups (*p <* 0.05). The average of the left forward stretching of the AOT significantly increased by 4.25 cm (*p <* 0.05), while the average of the control group significantly increased by 2.04 cm (*p <* 0.05) and there was a significant difference between the two groups (*p <* 0.05).

## 4. Discussion

Spasticity is the most common feature of cerebral palsy and occurs in approximately 85% of children with cerebral palsy, and correct assessment and treatment of spasticity are considered important. [20]. Most children with cerebral palsy spasticity have an asymmetrical body and disordered balance, leading to difficulties in daily activities [21]. In this study, changes in spasticity before and after training were confirmed by an ankle joint test and knee joint MTS. Curtis et al. (2014) [22] used an interactive dynamical stander to study low lateral flexor muscle spasticity of the ankle in children with cerebral palsy. Six children with cerebral palsy aged 4–10 years who were GMFCS level I–III were trained to activate ankle dorsi flexion for 10 weeks through ankle movements of the interactive dynamic stander of computer games. As a result, it was noted that the median active and passive dorsi flexion of the ankle increased by 5° and 10°, respectively; therefore, this training could function as a new clinical conservative treatment of ankle flexion in cerebral palsy children. In the right ankle joint contraction test in spasticity of ankle joint, both groups had a significant increase from before training to after training (*p <* 0.05), and in the left ankle joint contraction test, the AOT and control group increased from before training to after training (*p <* 0.05). Both groups’ range of motion of ankle was significantly increased because of symmetrical weight-bearing during the intervention and its stimulation of proprioceptive sensibility. Improvement in the range of motion of the ankle, which is a functional ankle movement, is possibly due to the voluntary forward, backward, left, and right weight shift movements of the lower extremity during the stage 1 to 3 in AOT. Increase in the range of motion of the ankle in the control group and the exercise program included sitting and standing up, weight-bearing, and weight movement training in the straight posture.

In the MTS used to examine the degree of spasticity, the right knee joint of the action observation training participants was 4.78° before training and 2.91° after training. The knee joint decreased from 3.77° before training to 1.91° after training, which is consistent with the results of previous studies and suggests that there is a connection between construction and rigidity. These results indicate that the range of motion of the ankle joint is increased with regards to ankle construction, and the action observation training program images provide the children to simultaneously watch the full-motion images and the enlarged images of the movements, which are an important part of the joint’s movement, were shown enlarged of children with diplegic cerebral palsy.

GMFM-88 is used as a most common tool to evaluate the function of children with cerebral palsy and down’s syndrome [23] by measuring and recording changes in exercise levels over time or over treatment outcomes [24]. In the current study, the B scale of the AOT was 93.33% before training and 98.45% after training, the C scale was 88.36% before training and 94.92% after training, the D scale was 60.68% before training and 77.48% after training, and the E scale was 46.26% before training and 52.77% after training. There was a significant difference before and after training (*p <* 0.05), and also in the B scale, C scale, D scale, and E scale in the control group (*p <* 0.05). This was supported by the results of the AOT group in this study since a more significant change in the GMFM-E results was observed compared to the control group. Although conducted in a relatively short amount of time, the repeated viewing of the videos (including shifting the body weight forward and backward), standing movements to improve balance in the third stage, and walking sideways in the fourth stage allowed for an easier understanding of the specific positions for each action and their order. This increased the exercise’s learning effects.

Park et al. reported [25] the effects of horseback riding treatment on gross motor function and functional performance in children with stiff cerebral palsy, and demonstrated a significant difference between the experimental group and the control group. Mahasup et al. studied [26] the effects of motor observation on 30 children with stiff diplegia cerebral palsy by applying action observation training for 2 months. In total, 15 control groups received general physical therapy that included the Bobath concept once a week, and 15 experimental groups performed action observation training three times a day; they demonstrated a significant difference in running and running and jumping between the two groups. The results of the current study are in agreement with previous studies and confirm that action observation training shows positive effects in improving GMFM. Therefore, action observation training improves the function of participants according to exercise level, and the action observation training was considered to contribute to the improvement of daily life skills and mobility of spastic bilateral lower limbs in cerebral palsy.

Children with cerebral palsy often have difficulty with sitting posture balance and have unstable postures, such as asymmetrical trunks and bending [27]. In addition, children with cerebral palsy have reduced movements in the trunk, pelvis, and lower extremities, so they stand and walk in unprepared condition, raising the upper extremities or excessively extending the upper bodies to compensate for insufficient antigravity activity [28]. Auld and Johnston (2014) [29] investigated the effects of an 8-week local community-based strengthening and balance exercise group on exercise in children with cerebral palsy. Five children with spastic diplegia cerebral palsy and five children with hemiplegia cerebral palsy participated in the study, and the results demonstrated that the participants’ balance ability increased significantly in Movement Assessment Battery for Children and anterior and lateral extension (*p <* 0.05). In the current study, the right side stretch of the AOT increased from 15.52 cm before training to 18.8 cm after training, the left side of the side stretch increased from 14.34 cm before training to 18.42 cm after training, the right side of the forward stretch increased from 22.78 cm before training to 27.38 cm after training, and the left side of the forward stretch increased from 22.6 cm before training to 26.85 cm after the training. The improvement of the arm stretch test indicates an improvement in balance, and it is thought that the movement observation training contributed to uniform weight bearing, postural alignment, and the ability to change direction by improving the muscles. There was also a significant difference before and after training in the two groups (*p <* 0.05); this may be due to the effect of action observation training on brain activity in the primary motor area, and the activation of cognitive activities related to motor memory formation and understanding of other people’s behavior through imitation [30].

This study has the following limitations: this study has a short 6-week intervention period, and this study comprises a small sample size. This makes it difficult to generalize the findings to all children with CP. It is also difficult to control all of the factors that might affect the child’s hormones affect because of puberty, scoliosis or hip problems, any influence of bracing on knee or ankle range of motion and spasticity, and activities of daily living. Furthermore, several of the participants had a short attention span while concentrating on the action observation, making it difficult for the treatment to last long as planned. This could be explained by the fact that children with spastic CP are not only impaired by the regulation ability of the muscular system and sensory deprivation, but are also deprived of cognitive function [31]. This study confirmed that action observation training has shown positive effects in improving the spasticity of ankle joint, gross motor function, and balance in children with cerebral palsy.

## Figures and Tables

**Table 1:** Action observation training protocol.

The first stage is to improve balance in the sitting position.					
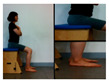	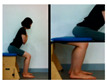	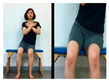	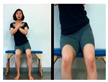	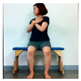	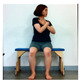
Upright the pelvis	Move weight forward	Move weight to the left and right		Rotate right and left	
The second stage is the sit-to-stand movement.					
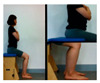		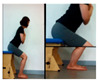		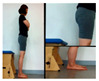	
Upright the pelvis in a sitting position		Move weight forward from a sitting position		Stand up from a sitting position	
The third stage is standing movements to improve balance.					
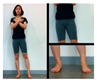	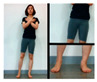	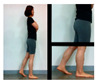	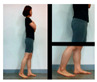	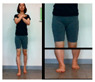	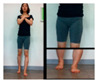
Move Weight Right and left		Forward weight shift with right foot and left foot (lateral view)		Forward weight shift with right foot and left foot (front)	
The fourth stage is walking sideways.					
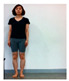	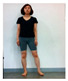		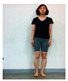	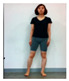	
Walking sideway to the left			Walking sideway to the right		

**Table 2:** General characteristics of subjects (*N* = 18).

Parameters	AOT (*n* = 9)	Control (*n* = 9)	t (*p*)
Gender, M/F (*n*, %)	3 (33.3)/6 (66.7)	5 (55.0)/4 (45.0)	1.141 (0.270)
Age (years)	7.44 ± 1.88 ^a^	6.90 ± 1.79	0.646 (0.527)
Height (cm)	122.60 ± 13.86	123.24 ± 14.18	−0.099 (0.922)
Weight (kg)	23.01 ± 6.71	27.73 ± 10.19	−1.176 (0.256)
GMFCS (I/II/III)	4/2/3	4/3/2	0.210 (0.837)

^a^ Mean ± SD; AOT: Action observation training; GMFCS: Gross motor function classification system.

**Table 3:** Differences in spasticity of ankle joint (*N* = 18).

Parameters		AOT (*n* = 9)	Control (*n* = 9)	t (*p*)
Ankle stiffness-right side (°)	Before	4.00 ± 4.72 ^a^	3.77 ± 4.87	−1.651 (0.118)
	After	10.58 ± 3.36	8.16 ± 3.58	
	Before-after	−6.58 ± 2.46	−4.39 ± 3.12	
	t(*p*)	−8.018 (0.000)	−4.223 (0.003)	
Ankle stiffness-left side (°)	Before	3.40 ± 4.92	3.34 ± 4.13	−1.411 (0.177)
	After	9.50 ± 3.45	7.71 ± 2.98	
	Before-after	−6.10 ± 2.76	−4.38 ± 2.41	
	t(*p*)	−6.632 (0.000)	−5.439 (0.001)	
MTS-right (kg)	Before	4.78 ± 3.26	3.57 ± 3.35	1.325 (0.204)
	After	2.91 ± 2.53	2.47 ± 2.99	
	Before-after	1.87 ± 1.46	1.09 ± 0.98	
	t(*p*)	−4.987 (0.001)	3.335 (0.010)	
MTS-left (kg)	Before	3.77 ± 1.46	4.29 ± 3.19	2.236 (0.040)
	After	1.91 ± 1.20	3.94 ± 3.28	
	Before-after	1.86 ± 1.80	0.34 ± 0.93	
	t(*p*)	3.093 (0.015)	1.106 (0.301)	

^a^ Mean ± SD; AOT: Action observation training; MTS: Modified Tardieu Scale.

**Table 4:** Differences in gross motor function (*N* = 18).

Parameters		AOT (*n* = 9)	Control (*n* = 9)	t (*p*)
**GMFM-B (%)**	Before	93.33 ± 6.07 ^a^	93.89 ± 5.40	−1.991 (0.064)
	After	98.45 ± 2.34	95.74 ± 4.72	
	Before-after	−5.12 ± 4.60	−1.85 ± 1.76	
	t(*p*)	−3.339 (0.010)	−3.162 (0.013)	
GMFM-C (%)	Before	88.36 ± 10.68	89.94 ± 9.88	−1.737 (0.102)
	After	94.92 ± 5.89	91.83 ± 8.49	
	Before-after	−6.56 ± 5.28	−2.89 ± 3.53	
	t(*p*)	−3.731 (0.006)	−2.449 (0.040)	
GMFM-D (%)	Before	60.68 ± 29.79	68.94 ± 23.02	−1.928 (0.072)
	After	77.48 ± 20.61	75.78 ± 21.79	
	Before-after	−16.80 ± 12.76	−6.84 ± 8.79	
	t(*p*)	−3.949 (0.004)	−2.334 (0.048)	
GMFM-E (%)	Before	46.26 ± 38.00	50.30 ± 32.82	−3.583 (0.002)
	After	52.77 ± 37.93	51.54 ± 32.26	
	Before-after	−6.51 ± 4.11	−1.23 ± 1.62	
	t(*p*)	−4.752 (0.001)	−2.285 (0.052)	

^a^ Mean ± SD; AOT: Action observation training; GMFM: Gross motor function measure.

**Table 5:** Differences in balance function (*N* = 18).

Parameters		AOT (*n* = 9)	Control (*n* = 9)	t (*p*)
PRT lateral-right (cm)	Before	15.52 ± 6.12 ^a^	14.94 ± 6.21	−2.327 (0.033)
	After	18.80 ± 6.42	16.33 ± 6.87	
	Before-after	−3.28 ± 1.71	−1.40 ± 1.72	
	t(*p*)	−5.751 (0.000)	−2.433 (0.041)	
PRT lateral-left (cm)	Before	14.34 ± 5.17	14.96 ± 6.68	−3.551 (0.003)
	After	18.42 ± 5.27	16.37 ± 6.75	
	Before-after	−4.08 ± 1.52	−1.42 ± 1.65	
	t(*p*)	−8.022 (0.000)	−2.578 (0.033)	
PRT frontal-right (cm)	Before	22.78 ± 7.16	20.35 ± 7.64	−2.154 (0.047)
	After	27.38 ± 7.81	22.37 ± 6.79	
	Before-after	−4.60 ± 2.87	−2.02 ± 2.16	
	t(*p*)	−4.811 (0.001)	−2.803 (0.023)	
PRT frontal-left (cm)	Before	22.60 ± 7.58	19.86 ± 7.54	−2.339 (0.033)
	After	26.85 ± 7.19	21.90 ± 7.41	
	Before-after	−4.25 ± 2.05	−2.04 ± 1.96	
	t(*p*)	−6.213 (0.000)	−3.116 (0.014)	

^a^ Mean ± SD; AOT: Action observation training; PRT: Pediatric reaching test.
